# Medical Societies in London; Discussion on Croup and Diphtheria; Barnes on the Use of Forceps in First Stage of Labor; Owen on Cleft Palate; Thornton on Drainage of the Abdominal Cavity

**Published:** 1879-07

**Authors:** C. T. Parkes

**Affiliations:** London


### Foreign Correspondence.

ARTICLE XII.

MEDICAL SOCIETIES IN LONDON. — DISCUSSION ON CROUP AND DIPHTHERIA. — BARNES ON THE USE OF FORCEPS IN FIRST STAGE OF LABOR. — OWEN ON CLEFT PALATE. — THORNTON ON DRAINAGE OF THE ABDOMINAL CAVITY.

LONDON, May 21, 1879.

Through the great courtesy shown me by the medical men whom I have met, it has been my privilege to be present during the sessions of several medical societies here, and to listen to the discussions upon several very important questions of practice, by the most noted men of the profession.

Most of these societies meet in the same room on Berners st.; the Pathological, the Medico-Chirurgical, the Obstetrical, and the Clinical, have their home in this hall. It is very comfortably fitted up, and ornamented with marble busts of departed presidents, so that the visitor's eye can wander over the features of the great and good men who accumulated the wealth of the past in the art of medicine and surgery, at the same time the visitor can sit near by and listen to the words of these other illustrious men who are busily investigating the facts of the present. The walls of the main rooms are crowded with books of every character, on all conceivable subjects, full doubtless of the ore into which the reader must delve and dig ere the crucible can contain the pure gold which lies at the bottom of the literary smelt. If man's ills and afflictions are in proportion to the books written about him, there should be no health in him, from the crown of his head to the soles of his feet. The books are kept well, and nicely and methodically arranged for reference. I admired, above all things, the evident determination to provide for the physical comfort of the members, by keeping everything neat and cosy. Best of all was the jolly social seance after the discussion, over a cup of coffee and a sandwich. The closing hour is ten o'clock, and about that time the tempting aroma from the steaming cups floats through the rooms, tickling the nostrils and stimulating the salivaries. What man could be mean enough to continue to speak in the midst of all this, even if he were wound up? It constitutes the gentlest and most seductive hint imaginable to discontinue a paper or discussion.

The cordiality with which strangers are received is beyond all praise. After the names have been presented to the president, by the secretary, the former officer formally welcomes them into the society as members for that session, and they are entitled to all the privileges of Fellows; clear through to coffee. One is made to feel at home; what more could be done? I have heard a good deal said about English exclusiveness and coldness. I don't believe a true English gentleman has any such attribute belonging to him, they are the most generous, the most charmingly hospitable people one can ever meet in the way of accommodation and good will. The only thing is to get right down to this deep stratum and be worthy of it.

They don't like prosy discussions at these meetings; if any unfortunate indulges in a "class meeting" experience, without very much direct application to the subject in hand, the rather cold-blooded shuffling of feet places the icy grasp of death upon his flow of eloquence or otherwise, and he is thankful for the opportunity to resume his seat. The bane of all medical societies has not been entirely eliminated in these well regulated assemblies. It is truly marvellous how speakers with "wonderful" cases, still more wonderfully *àpropos*, will spring up in every discussion imaginable, and I presume in all quarters of the globe. Then, too, it is just as usual to find what I am loath to call the orchestrion of medical debates. This instrument, you must know, is a good sized box containing a brass band (40 pieces), an organ, a bagpipe, etc., so arranged that by the touch of a spring and the turn of a crank, one can revel in the master pieces of Bethoven, Straus, Handel et al., without worry or an expenditure of guineas. Now, these medical "orchestrions" are good things to have on hand when there is nothing to do but to listen to words without end. When one desires to learn the curt experiences of thinking men on vital issues, they are fearfully exasperating. A speech of an hour thus yields but a minute's gist. Perhaps such discussions are too much impromptu in their character to allow of generalization. I do not know that they can be changed for the better. When a man talks rather well and readily, he is about as apt to know of his talent as any one else, and it is certainly hard to ask him to hide that which he owns under a bushel. So let us be generous; ramble on, sweet talkers, you amuse if you do not interest.

The debate on the similarity or non-similarity, pathologically, of membranous croup and diphtheria, grew out of the report of a committee of the ablest men in the profession, appointed some time ago, to inquire into those important questions. It was held at the sessions of the Medico-Chirurgical Society, and such men as Dr. Johnson, Mr. Parker, Mr. Semple, Sir Wm. Gull, Sir Wm. Jenner, Mr. Jonathan Hutchinson and Mr. Wilks took part in the discussion upon the report. In the main the illustrious speakers evinced the usual proclivity to "agree to disagree," some showing a marked tendency to do more of the latter than the former. A full report of the remarks made can be found in the *Lancet* of April 26th and May 3d and 17th. There seems to be a growing tendency to admit a close relationship between the two maladies even by men who have hitherto held valiantly to the belief that they were totally distinct and separate from each other.

The principal element of disagreement came from such men as Mr. Jonathan Hutchinson and Dr. Wilks. The sum of Mr. Hutchinson's remarks are embraced in these inquiries: "Is there any *bona fide* and sound reason for speaking of diphtheria as a specific fever? Is it not much more probable that the diphtheritic false membrane may arize from *ordinary causes* of *inflammation*, and that it becomes itself contagious?" It had no period of incubation. He was inclined to believe with Sir John Cormack, heading his lectures "croup, a symptom; diphtheria, a disease." This idea of the ordinary origin of diphtheria and its auto-inoculability was moderately acquiesced in by Sir Wm. Gull, a man whose *sang froid* is absolutely inimitable. It was rather startling to hear him say that the results of his experience had forced him to the belief that many of these cases were benefited by resort to the discarded mercurial treatment. If I remember aright, just before I left home, an article was going the rounds of the journals, in which a practitioner in the Eastern States claimed to cure all his cases by 02.5 Gm. doses of calomel. I hope it will please him to know that he has good authority at his back. Sir Wm. Jenner could find no ground upon which to rest his feet except that furnished by the assumption that both affections were "*one and the same.*" Mr. Semple stands on this ground, and Dr. Johnson finds all other very slippery.

As the conclusions of the committee may interest you, I send them.

1. Membranous inflammation confined to, or chiefly affecting the larynx and trachea, may arise from a variety of causes, as follows:

(*a.*)—From diphtheritic contagion.

(*b.*)—By means of foul water or foul air, or other agents such as are commonly concerned in the generation or transmission of zymotic disease (though whether as mere carriers of disease cannot be determined).

(*c.*)—As an accompaniment of measles, scarlatina or typhoid, being associated with these diseases independently of any ascertainable exposure to the special diphtheritic infection.

(*d.*)—It is stated on apparently conclusive evidence, although the committee have not had an opportunity in any instance of examining the membrane in question, that membranous inflammation of the larynx and trachea may be produced by various accidental causes of irritation, the inhalation of hot water or steam, the contact of acid, the presence of a foreign body in the larynx and a *cut throat.*

2. There is evidence in cases which have fallen under the observation of members of the committee and are mentioned in the tables appended, that membranous affection of the larynx and trachea has shortly followed exposure to cold, but their knowledge of the individual cases is not sufficient to exclude the possible intervention or co-existence of other causes. The majority of cases of croupal symptoms definitely traceable to cold, appear to be of the nature of laryngeal catarrh.

3. Membranous inflammation, chiefly of the larynx and trachea, to which the term "membranous croup" would commonly be applied, may be imparted by an influence, epidemic or of other sort, which has in other persons produced *pharyngeal* diphtheria.

4. And conversely, a person suffering with the membranous affection chiefly of the air passages, such as would commonly be termed membranous croup, may communicate to another a membranous condition limited to the pharynx and tonsils, which will be commonly regarded as diphtheritic. * * * The membrane, even when chiefly laryngeal, is more often than not associated with some extent of a similar change in the pharynx or on the tonsils; and whether we have regard to the construction of the membrane, or to the constitutional state, as evinced by the presence of albumen in the urine, it is not practicable to show an absolute line of demarcation (save what depends upon the position of the membrane) between the pharyngeal and laryngeal forms of the disease.

The facts before the committee only warrant them in the view, that when it obviously occurs from a zymotic cause or distinct infection and primarily affects the pharynx, constitutional depression is more marked, and albuminuria more often and more largely present; though in both conditions some albumen in the urine is more frequently present than absent. The committee suggest that the term *croup* be henceforth used wholly as a clinical definition implying laryngeal obstruction, occurring with febrile symptoms in children. Thus croup may be membranous or not membranous, due to diphtheria or not so.

The term *diphtheria* is the anatomical definition of a zymotic disease which may or may not be attended with croup."

The report certainly covers enough ground to warrant the prolonged discussion which it awakened and which is not yet finished. The opinions expressed cannot fail to be very interesting and full of valuable information. The subject of treatment was scarcely alluded to by any one but Sir Wm. Gull; between the mercurials suggested by him and the alcohol insisted upon by Dr. Chapman, of the States, no doubt lies the safety, and perhaps restoration to health of the sorely afflicted.

I heard the commencement of a very interesting discussion at the meeting of the Obstetrical Society, on the use of the forceps in the first stages of labors, in "lingering" and "tedious" cases. The debate was opened by the renowned Dr. Barnes, and his address was marked by his usual perspicuity and wisdom. I am sorry that I have not the space to send you his conclusions. Suffice it to say that he justified their use in preference to *ergot* or *waiting*, in the specified cases of no recognizable obstruction and purely "lingering" character.

I was somewhat amused to hear Mr. Thorburn, of Manchester, describe his efforts to make the English answer as well as the American forceps for purposes of delivery. He told how he had been compelled to resort to considerable mechanical ingenuity in the fitting of screws in the handles, etc., to increase their compressive power and prehensile capacity. The idea never seemed to enter his head that it would have been much simpler and easier to have used the American forceps at once—the natural sequence of their acknowledged superiority. One gentleman certainly displayed praiseworthy courage in reporting the results of ten years' practice in midwifery. As the reports of the society in the *Lancet*, May 17th, contain these statistics as furnished, I can properly refer to them. It is certainly an astounding record of "still births," in part acknowledged as being attributable to the use of ergot. If any one is ever justified in blindly accepting a dictum, these statistics surely suggest that the one which follows, should be written upon the walls of every obstetrical theater; "never use ergot in the parturient chamber until the womb is emptied of its contents."

One of the newest and to me most interesting works to which attention has been called, is in the line of dentistry, and done by an American, Dr. W. F. Thompson, formerly of Chicago. Teeth are removed, kept out of the mouth, well covered in warm absorbent cotton, sufficiently long to fill the largest cavity, or even to build up an entire crown of gold, and then returned to their proper cavity in the jaw. I have seen the cases and know that the tooth has become as fast and as useful as any. The operation is only applicable to such teeth as cannot be treated in the mouth, owing to the extent of decay. The success seems to follow great care in cleansing the tooth socket of blood clots, maintaining at a certain degree the warmth of the tooth, and above all, providing for drainage of the socket for a few days after the tooth is replaced. This is accomplished by using a rather thick-walled gold tube introduced to the bottom of the tooth cavity, around which the filling is fixed. After the tube has acccomplished its purpose and the tooth become fastened and free from tenderness, the tube is closed. The latter is peculiarly the doctor's own method.

A visit to the Harveian Society gave me the pleasure of listening to a splendid paper on cleft palate, read by Mr. E. Owen. It was exceedingly well written and full of interest. The doctor concludes that the operation is followed by the most successful results if done about the third year of life. If the cleft is complete, it is best done in three stages. 1st, close the hare-lip, and this is followed by some contraction of the opening in the hard palate. 2d, close the fissure in the soft palate, this again is succeeded by still more diminution of the size of the opening in the bony portion. 3d, operate for the closure of the opening in the hard palate. Anæsthetics should be used in all cases, and Mr. Smith's gag is the best one he has ever employed for keeping the jaws well open. He has avoided all hæmorrhage in the final division of the muscles of the soft palate successfully, by making the section with the small slender point of Pacquelin's thermo-cautery. The greatest of care must be taken of the little patients after the operation, to amuse them just to the point short of laughing, and to carefully keep them above the worry which might end in crying. Rest, absolute and perfect, of the parts operated upon must be sedulously sought after and secured. They should be schooled somewhat to the confinement necessary after the operation for several days previous to its performance.

The subject of greatest interest to me at this meeting was treated of in the paper read by Mr. Knowsley Thornton, surgeon to the Samaritan Hospital for Women, on drainage in abdominal surgery. The deductions and conclusions were based mainly upon cases of ovarian tumor, but are, as claimed by Mr. T., of universal application to all cutting involving the abdominal walls. Mr. Thornton has evidently gone through two stages of belief with reference to the usefulness of drainage in these cases, and is now emerging upon a third. 1st. Drainage seemed to give the very best of results, when a bad case or so, explainable apparently only on the ground of harmful irritation arising from the tube itself, led him to dispense with it. 2d. Quite a number of successful cases without any drainage whatever, after rather formidable operations, have led him to his present impression that with truly honest and efficient antiseptic operations, the cases requiring any drainage whatever will be very few indeed. A well closed abdominal wall after the operation is, in his mind, the safest condition for the patient to be in—a rule with few exceptions. He insists on the absolute necessity of using antiseptics, and his great experience make his utterances authoritative. Mr. Thornton is a very lucid, earnest writer, and close thinker. With over one hundred cases done by himself, with an unusually small mortality, and these added to his careful deduction, from the hundreds of cases in which he has been Mr. Spencer Wells' main and only assistant, the results of his experience are most valuable to the profession.

His readiness as an operator, his mastery of the pathology of the entire subject, his wealth of clinical study and observation, prognosticate for him a position in this field of valuable labor, unsurpassed by any who have worked in it in the past or whose labors are devoted to it in the present. If I felt at liberty so to do, I could give you an instance of the man's coolness, courage and readiness of resource under trying circumstances, which would convince you that he has already reached a climax beyond which it is impossible for any man to be taxed.

I have been intending to write you a letter about the ovariotomies I have seen while in London, but Mr. Thornton and his colleague, Dr. Bantock, do the operation so perfectly and with such elegance that I do not dare to make the attempt until I feel myself more securely grounded. Their skill of operating is only surpassed by the watchful care given to any case until recovery is insured. I will write more thereon some other time.

Following the beaten track, I have spent a few stray hours in Westminster Abbey, only to be disgusted at the neglect heaped upon the medical profession. A forest of monuments fill its naves and chapels erected to soldiers and sailors, from the foretop-man to the commodore, from the corporal to the Iron Duke. Preachers are built up in marbled abundance, poets are chiseled in solid verse, lawyers are briefly done in stone, but the doctors are nowhere. This almost leads one to believe that doctors are the only people who do not die in this country. Among other things noticed (under this oldest of roofs), was a piece of marble in wretched repair; it was labelled, "to the memory of an honest man." Even he died.

It is susceptible of proof that doctors do not kill any more people than soldiers. They certainly ought to receive acknowledgment of what is accomplished; especially, as what *is* done in that way, is done with much less noise than that made by the red coats.

More courage, self-sacrificing promptitude of action, generosity of spirit, true manliness and honesty are displayed every day in these streets by professional men in their ordinary rounds of duty than was ever thought of by so-called geniuses that have chimed a "well told tale," or fought a "hard won battle."

Poets as often sicken the mind as doctors do the body; then why a poet's corner while the doctor's surgery is passed by in silence? Here is a widely opened field for the famed English "fair play" to work wonders. The only name I saw here followed by the title of doctor, was that of Oliver Goldsmith, but as he, so far as I know, is only noted in a medical way for having held a learned and convincing conversation with Boswell on the movements of the upper jaw in the process of mastication. Good man as he was, it is hardly fair to make him the representative among the dead of the profession which has to do with the science and art of medicine and surgery. I do not expect to be found resting in Westminster Abbey in the future, unless this wretchedly prolix letter entitles me to a slab.

C. T. PARKES.

